# Supplementary material for: Ultrafast data mining of molecular assemblies in multiplexed high-density super-resolution images
Source: Nat Commun. 2019 Jan 10;10:119. doi: 10.1038/s41467-018-08048-2 (PMC6328550; doi:10.1038/s41467-018-08048-2)
Supplement: Supplementary file 1 — Supplementary Information [file 41467_2018_8048_MOESM1_ESM.pdf]

Ultrafast Data Mining of Molecular Assemblies in Multiplexed High-Density Super-Resolution Images

Yin et al.

Supplementary Discussion

Given the sparse nature of SMLM data, one major advantage of dTC as compared to the ftTC algorithm is its improved computational speed. Specifically, since the ftTC is calculated via the bispectrum convolution theorem its computational expenditure reaches $\sim(N \log_2 N)^4$ for 4D Fourier Transform, but in practice it would require an outflow of $\sim(N^2 \cdot N \log_2 N)^2$ due to the insufficient capacity of RAM (see ref 20). In contrast, the dTC algorithm visits each of the SMLM coordinates -- which are much less than N^2 due to their sparsity, and partitions three measures (the distances from the visited coordinate to other coordinates in the other two channels, and the angle $\Delta\theta$ in between each pairwise vectors) into 3-D bins (**Supplementary Note 1**). Because the user-defined bins do not have to be evenly sampled and can only focus on the configurations of interests with high sampling frequency, such tri-variate histogram bin counting further saves the computation costs. We note that this dTC algorithm can also be adapted to the calculation of the Pair-Correlation (dPC) function, although the 2D-Fourier Transform based computation of the Pair-Correlation (ftPC) function is already fast enough in most situations (**Supplementary Note 2**). We provided Matlab-executable function for Pair-Correlation computing via the dPC algorithm (**Supplementary Note 2**, **Supplementary Software**).

We also note that implementation of GPU (NVIDIA GTX 1060) does not improve the computation performance in calculating the Triple-Correlation function via dTC (Fig. 2C). As mentioned above, the essence of the dTC algorithm is the tri-variate (3-D) histogram bin counting during visiting each coordinate. Since the number of the 3-D sampling bins is usually larger than the capacity of the shared memory of most of the current GPU, the histogram bin counting cannot be decomposed into smaller data blocks for parallel computation. Figure 2C shows the evaluation of the naive 3-D histogramming on the global memory of the GPU, indicating no improvement in computation performance as compared to performance on CPU.

Supplementary Note 1

Since the SMLM imaging generates a list/set of coordinates $\mathbb{C} = \{\mathbf{r}_i\}$ where the molecules localize, an SMLM image can be described as $f(\mathbf{R})$ where $f(\mathbf{R}) = 1$ $(\mathbf{R} \in \mathbb{C})$ and $f(\mathbf{R}) = 0$ $(\mathbf{R} \notin \mathbb{C})$. Considering a three-color SMLM image $f_{\mathrm{CH1}}(\mathbf{R})$, $f_{\mathrm{CH2}}(\mathbf{R})$, and $f_{\mathrm{CH3}}(\mathbf{R})$, with its single-molecule localization coordinate sets $\mathbb{C}_{\mathrm{CH1}} = \{\mathbf{r}_i^{\mathrm{CH1}}\}$, $\mathbb{C}_{\mathrm{CH2}} = \{\mathbf{r}_i^{\mathrm{CH2}}\}$, and $\mathbb{C}_{\mathrm{CH3}} = \{\mathbf{r}_i^{\mathrm{CH3}}\}$ from three different color channels CH_1, CH_2, and CH_3, its triple-correlation function is defined as $g(\mathbf{r}_{12}, \mathbf{r}_{13}) = \langle \rho_{\mathrm{CH1}}(\mathbf{R}) \rho_{\mathrm{CH2}}(\mathbf{R} + \mathbf{r}_{12}) \rho_{\mathrm{CH3}}(\mathbf{R} + \mathbf{r}_{13}) \rangle_{\mathbf{R}} \ / \ (\langle \rho_{\mathrm{CH1}}(\mathbf{R}) \rangle_{\mathbf{R}} \langle \rho_{\mathrm{CH2}}(\mathbf{R}) \rangle_{\mathbf{R}} \langle \rho_{\mathrm{CH3}}(\mathbf{R}) \rangle_{\mathbf{R}})$ where $\langle \quad \rangle_{\mathbf{R}}$ denotes averaging over all positions $\mathbf{R}$ in the image; $\rho(\mathbf{R}) = \left(\int_{\Delta S} f(\mathbf{R})\right) / \Delta S$ is the local density at $\mathbf{R}$ within a differential area ΔS (**Fig. 1a**). Given the fact that $\rho_{\mathrm{CH1}}(\mathbf{R}) \rho_{\mathrm{CH2}}(\mathbf{R} + \mathbf{r}_{12}) \rho_{\mathrm{CH3}}(\mathbf{R} + \mathbf{r}_{13}) \equiv 0$ $(\mathbf{R} \notin \mathbb{C})$, the triple-correlation function can be derived as Supplementary Equation (1) :

$$g(\mathbf{r}_{12}, \mathbf{r}_{13}) = \frac{1}{\rho_1 \rho_2 \rho_3} \cdot \frac{1}{A} \sum_i \rho_{\mathrm{CH2}}\left(\mathbf{r}_{12}^{(i)}\right) \rho_{\mathrm{CH3}}\left(\mathbf{r}_{13}^{(i)}\right) \quad (1)$$

where A and $\rho_i = \langle \rho(\mathbf{R}) \rangle_{\mathbf{R}}$ is the image size and the average density of the entire SMLM image in the i-th channel, respectively; $\rho_{\mathrm{CH}_l}\left(\mathbf{r}_{1l}^{(i)}\right)$ represents the local density at $\mathbf{r}$ in CH_l originated from the i-th coordinates in CH_1. Moreover, since in most situations the orientation of each triplet $\{\mathbf{r}_i^{\mathrm{CH1}}, \mathbf{r}_j^{\mathrm{CH2}}, \mathbf{r}_k^{\mathrm{CH3}}\}$ is independent of that of the whole image, $g(\mathbf{r}_{12}, \mathbf{r}_{13}) = g(r_{12}, \theta_{12}, r_{13}, \theta_{13} = \theta_{12} + \Delta\theta)$ is further averaged along θ_{12} from 0 to 2π (**Fig. 1a**), and derived as Supplementary Equation (2):

$$g(r_{12}, r_{13}, \Delta\theta) = \frac{1}{2\pi A \rho_1 \rho_2 \rho_3} \sum_i \int_0^{2\pi} \mathrm{d}\theta \rho_{\mathrm{CH2}}\left(r_{12}^{(i)}, \theta^{(i)}\right) \rho_{\mathrm{CH3}}\left(r_{13}^{(i)}, (\theta + \Delta\theta)^{(i)}\right) \quad (2)$$

We note that the integration through $[0,2\pi)$ at distance r from $\mathbf{r}_i$ could exceed the image area, and in such cases, integration has to be limited within $\theta \in [\varphi_1, \varphi_2)$ $(0 \leq |\varphi_2 - \varphi_1| \leq 2\pi)$, where all $\theta \notin (\varphi_1, \varphi_2)$ has to meet such condition that at least one of $\mathbf{r}_{12} = (r_{12}, \theta)$ and $\mathbf{r}_{13} = (r_{13}, \theta + \Delta\theta)$ is outside the image area (**Supplementary Fig. 1**).

Given the binary nature of an SMLM image $f(\mathbf{R})$, the derivation provided above describes a direct TC algorithm (dTC) for computing $g(r_{12}, r_{13}, \Delta\theta)$: 1) visiting a given detection at $\mathbf{r}_i$ in CH_1 and assigning it as origin O; 2) calculating $\rho_{\mathrm{CH2}}(r_{12}, \theta)$ and $\rho_{\mathrm{CH2}}(r_{13}, \theta + \Delta\theta)$; 3) averaging such calculations in 2) along θ (**Fig. 1a**); and 4) iteratively visiting every coordinate in CH_1 and repeating 1) - 3) for each visit (**Fig. 1b**). The calculated $g(r_{12}, r_{13}, \Delta\theta)$ is then mapped to $g(r_{12}, r_{13}, r_{23})$.

We note that the Pair-Correlation can be defined as $g(\mathbf{r}) = \langle \rho_{\mathrm{CH1}}(\mathbf{R}) \rho_{\mathrm{CH2}}(\mathbf{R} + \mathbf{r}) \rangle_{\mathbf{R}} / (\langle \rho_{\mathrm{CH1}}(\mathbf{R}) \rangle_{\mathbf{R}} \langle \rho_{\mathrm{CH2}}(\mathbf{R}) \rangle_{\mathbf{R}})$ or $g_\delta(\mathbf{r}) = \langle \delta\rho_{\mathrm{CH1}}(\mathbf{R}) \delta\rho_{\mathrm{CH2}}(\mathbf{R} + \mathbf{r}) \rangle_{\mathbf{R}} / (\langle \rho_{\mathrm{CH1}}(\mathbf{R}) \rangle_{\mathbf{R}} \langle \rho_{\mathrm{CH2}}(\mathbf{R}) \rangle_{\mathbf{R}})$ where $\delta\rho_{\mathrm{CH1}}(\mathbf{R}) = \rho_{\mathrm{CH1}}(\mathbf{R}) - \langle \rho_{\mathrm{CH1}}(\mathbf{R}) \rangle_{\mathbf{R}}$. These two definitions are both accepted and applied because $g(\mathbf{r}) \equiv g_\delta(\mathbf{r}) + 1$. Similarly, the Triple-Correlation function can be also defined in two forms. Besides the definition given in this work, it can be defined as $g_\delta(\mathbf{r}_{12}, \mathbf{r}_{13}) =$

$\langle\delta\rho_{\mathrm{CH1}}(\mathbf{R})\delta\rho_{\mathrm{CH2}}(\mathbf{R}+\mathbf{r}_{12})\delta\rho_{\mathrm{CH3}}(\mathbf{R}+\mathbf{r}_{13})\rangle_{\mathbf{R}}/(\langle\rho_{\mathrm{CH1}}(\mathbf{R})\rangle_{\mathbf{R}}\langle\rho_{\mathrm{CH2}}(\mathbf{R})\rangle_{\mathbf{R}}\langle\rho_{\mathrm{CH3}}(\mathbf{R})\rangle_{\mathbf{R}})$ [1]. However, the difference between these two definitions is not a constant scaler.

$$\begin{aligned} g(\mathbf{r}_{12},\mathbf{r}_{13}) &= \langle(\delta\rho_{\mathrm{CH1}}(\mathbf{R})+\rho_1)(\delta\rho_{\mathrm{CH2}}(\mathbf{R}+\mathbf{r}_{12})+\rho_2)(\delta\rho_{\mathrm{CH3}}(\mathbf{R}+\mathbf{r}_{13})+\rho_3)\rangle_{\mathbf{R}}/(\rho_1\rho_2\rho_3) \\ &= \langle\delta\rho_{\mathrm{CH1}}(\mathbf{R})\delta\rho_{\mathrm{CH2}}(\mathbf{R}+\mathbf{r}_{12})\delta\rho_{\mathrm{CH3}}(\mathbf{R}+\mathbf{r}_{13})\rangle_{\mathbf{R}}/(\rho_1\rho_2\rho_3) + \langle\delta\rho_{\mathrm{CH1}}(\mathbf{R})\delta\rho_{\mathrm{CH2}}(\mathbf{R}+\mathbf{r}_{12})\rangle_{\mathbf{R}}/(\rho_1\rho_2) \\ &\quad + \langle\delta\rho_{\mathrm{CH2}}(\mathbf{R}+\mathbf{r}_{12})\delta\rho_{\mathrm{CH3}}(\mathbf{R}+\mathbf{r}_{13})\rangle_{\mathbf{R}}/(\rho_2\rho_3) + \langle\delta\rho_{\mathrm{CH1}}(\mathbf{R})\delta\rho_{\mathrm{CH3}}(\mathbf{R}+\mathbf{r}_{13})\rangle_{\mathbf{R}}/(\rho_1\rho_3) + 3 \\ &= g_\delta(\mathbf{r}_{12},\mathbf{r}_{13}) + g_{12}(\mathbf{r}_{12}) + g_{13}(\mathbf{r}_{13}) + g_{23}(\mathbf{r}_{23}) \end{aligned}$$

where $g_{\mathrm{XY}}(\mathbf{r}_{\mathrm{XY}})$ is the Cross-Pair-Correlation $g_{\mathrm{XY}}(\mathbf{r}_{\mathrm{XY}}) = \langle\rho_{\mathrm{CHX}}(\mathbf{R})\rho_{\mathrm{CHY}}(\mathbf{R}+\mathbf{r}_{\mathrm{XY}})\rangle_{\mathbf{R}}/(\langle\rho_{\mathrm{CHX}}(\mathbf{R})\rangle_{\mathbf{R}}\langle\rho_{\mathrm{CHY}}(\mathbf{R})\rangle_{\mathbf{R}})$. The derivation demonstrates that $g(\mathbf{r}_{12},\mathbf{r}_{13})$ results in a mixture of trimer and dimer correlations while $g_\delta(\mathbf{r}_{12},\mathbf{r}_{13})$ only contains the trimer correlation (Supplementary Fig. 2), which is in fact the most of our interests. Since the dTC algorithm presented in this work follows the first kind definition of the Triple-Correlation function $g(\mathbf{r}_{12},\mathbf{r}_{13})$, we extract the Cross-Pair-Correlation from the dTC results to generate the final $g_\delta(\mathbf{r}_{12},\mathbf{r}_{13})$ profile (Supplementary Fig. 2).

To further interpret the triple-correlation results, we analyzed the local maxima of $g(r_{12},r_{13},r_{23})$, which represent the most significant geometric configuration defined by $\{r_{12},r_{13},r_{23}\}$ amongst the three colors. The configurations are then displayed as triangles in which the size of the circle at the vertex denotes the correlation amplitude (**Fig. 1d**).

Supplementary Note 2

The coordinate-visiting algorithm presented in this work for Triple-Correlation can be adapted to computing the Pair-Correlation function. Considering a two-color SMLM image $f_{\mathrm{CH1}}(\mathbf{R})$ and $f_{\mathrm{CH2}}(\mathbf{R})$ (can be "two" same channels for Auto-Pair-Correlation), with its single-molecule localization coordinate sets $\mathbb{C}_{\mathrm{CH1}} = \{\mathbf{r}_i^{\mathrm{CH1}}\}$ and $\mathbb{C}_{\mathrm{CH2}} = \{\mathbf{r}_i^{\mathrm{CH2}}\}$ from two color channels CH_1 and CH_2, its Pair-Correlation Function is defined as $g(\mathbf{r}) = \langle \rho_{\mathrm{CH1}}(\mathbf{R})\rho_{\mathrm{CH2}}(\mathbf{R}+\mathbf{r})\rangle_{\mathbf{R}} \;/\; (\langle \rho_{\mathrm{CH1}}(\mathbf{R})\rangle_{\mathbf{R}} \langle \rho_{\mathrm{CH2}}(\mathbf{R})\rangle_{\mathbf{R}})$ where $\langle \quad \rangle_{\mathbf{R}}$ denotes averaging over all positions $\mathbf{R}$ in the image; $\rho(\mathbf{R}) = \left(\int_{\Delta S} f(\mathbf{R})\right)/\Delta S$ is the local density at $\mathbf{R}$ within a differential area ΔS. Given the fact that $\rho_{\mathrm{CH1}}(\mathbf{R})\rho_{\mathrm{CH2}}(\mathbf{R}+\mathbf{r}) \equiv 0 \ (\mathbf{R} \notin \mathbb{C})$, the Pair-Correlation function can be derived as Supplementary Equation (3):

$$g(\mathbf{r}) = \frac{1}{\rho_1\rho_2} \cdot \frac{1}{A}\sum_i \rho_{\mathrm{CH2}}\left(\mathbf{r}^{(i)}\right) \tag{3}$$

where A and $\rho_i = \langle \rho(\mathbf{R})\rangle_{\mathbf{R}}$ is the image size and the average density of the entire SMLM image in the i-th channel, respectively; $\rho_{\mathrm{CH2}}\left(\mathbf{r}^{(i)}\right)$ represents the local density at $\mathbf{r}$ in CH_2 originated from the i-th coordinates in CH_1. Moreover, since in most situations the orientation of each pair $\{\mathbf{r}_i^{\mathrm{CH1}}, \mathbf{r}_j^{\mathrm{CH2}}\}$ is independent of that of the whole image, $g(\mathbf{r}) = g(r,\theta)$ is further averaged along θ from φ_1 to φ_2, and derived as Eq(4)

$$g(r) = \frac{1}{|\varphi_2 - \varphi_1| A\rho_1\rho_2}\sum_i \int_{\varphi_1}^{\varphi_2} \mathrm{d}\theta \rho_{\mathrm{CH2}}\left(r^{(i)}, \theta^{(i)}\right) \tag{4}$$

where $0 \le |\varphi_2 - \varphi_1| \le 2\pi$ must satisfy that $\mathbf{r} = \left(r, \theta \middle| \theta \notin (\varphi_1, \varphi_2)\right)$ is outside the image (similar to Supplementary Fig. 1). This direct Pair-Correlation (dPC) algorithm can be proceeded as following: 1) visiting a given detection at $\mathbf{r}_i$ in CH_1 and assigning it as origin O; 2) calculating ρ_{CH2} within part of a ring from φ_1 to φ_2 at $r + \mathrm{d}r$; and 3) iteratively visiting every coordinate in CH_1 and repeating 1) -2) for each visit.

Similarly, this dPC algorithm achieves faster performance than FT based convolution theorem. The former depends on the number of localizations in Channel 1 and the number of sampling bins of r, whereas the latter depends on the number of pixels in the entire image. One could accelerate the FT based PC calculation by either decreasing the size of the input image or enlarging its pixel size, but again the accuracy of the calculation would be compromised accordingly. On the other hand, the dPC can be performed faster by tuning the user-defined sampling bins, namely, since the sampling bins do not have to be evenly distributed, one can sample r with a higher frequency for distances having more biological relevance and with a lower frequency for distances with lower relevance. Additionally, the Auto-PC can be used to describe the localization precision[2]. In this scenario, one might want to profile the Auto-PC function with high frequency at shorter distances (e.g. every 1 nm within [0, 10 nm]) to fit the localization accuracy which is usually 5 – 15 nm[3]. For this purpose, the FT based calculation of the Pair-Correlation function requires to render the SMLM data into pixels of 1×1 nm^2, and perform 2D Fourier Transform on numerous pixels accordingly, whereas the dPC algorithm presented here can be performed more

efficiently by tuning the sampling bins with 1 nm^{-1} frequency at shorter distances but lower frequency at longer distances.

The executable Matlab function `smPairCorrelation.m` in the Supplementary Software can be used to compute the Pair Correlation function via the dPC algorithm. The input parameter `RhoUpper` is the upper edges of the user defined bins, which has to be of the same unit as other input parameters (coordinates and image sizes).

Supplementary Figures 1 - 6

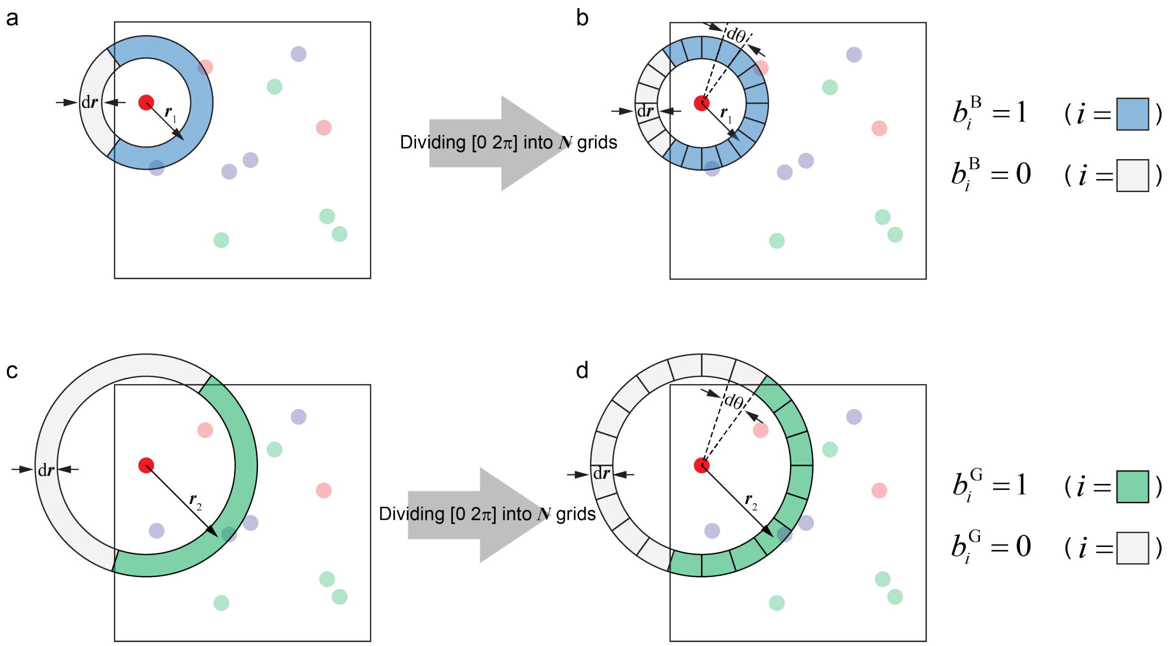

Supplementary Figure 1. | (**a** and **c**). As mentioned the main text in calculating Supplementary Equation (2), the integration through $0 \leq |\varphi_2 - \varphi_1| \leq 2\pi$ (blue area in (a) and (b); green area in (c) and (d)), has to make sure that all possible $\theta \notin (\varphi_1, \varphi_2)$ affirms at least one of $\mathbf{r}_{12} = (r_{12}, \theta)$ and $\mathbf{r}_{13} = (r_{13}, \theta + \Delta\theta)$ is outside the image area. (**b** and **d**). To determine $[\varphi_1, \varphi_2]$, again, we gridded $[0, 2\pi]$ with $\mathrm{d}\theta = \pi/45$, and scored each grid as $b_i^{\mathrm{B}} = 1$ ($b_i^{\mathrm{G}} = 1$) at $r = r_{12}$ ($r = r_{13}$) if inside the canvas, or $= 0$ if otherwise. The 'area' at a given $(r_{12}, r_{13}, \Delta\theta)$ was then calculated as $S = \sum_i b_i b_{i+\Delta\theta} (r_{12} \mathrm{d}r \mathrm{d}\theta)(r_{13} \mathrm{d}r \mathrm{d}\theta)$, where $b_{i+\Delta\theta}$ stands for the jth grid that $\Delta\theta$ away from the ith one.

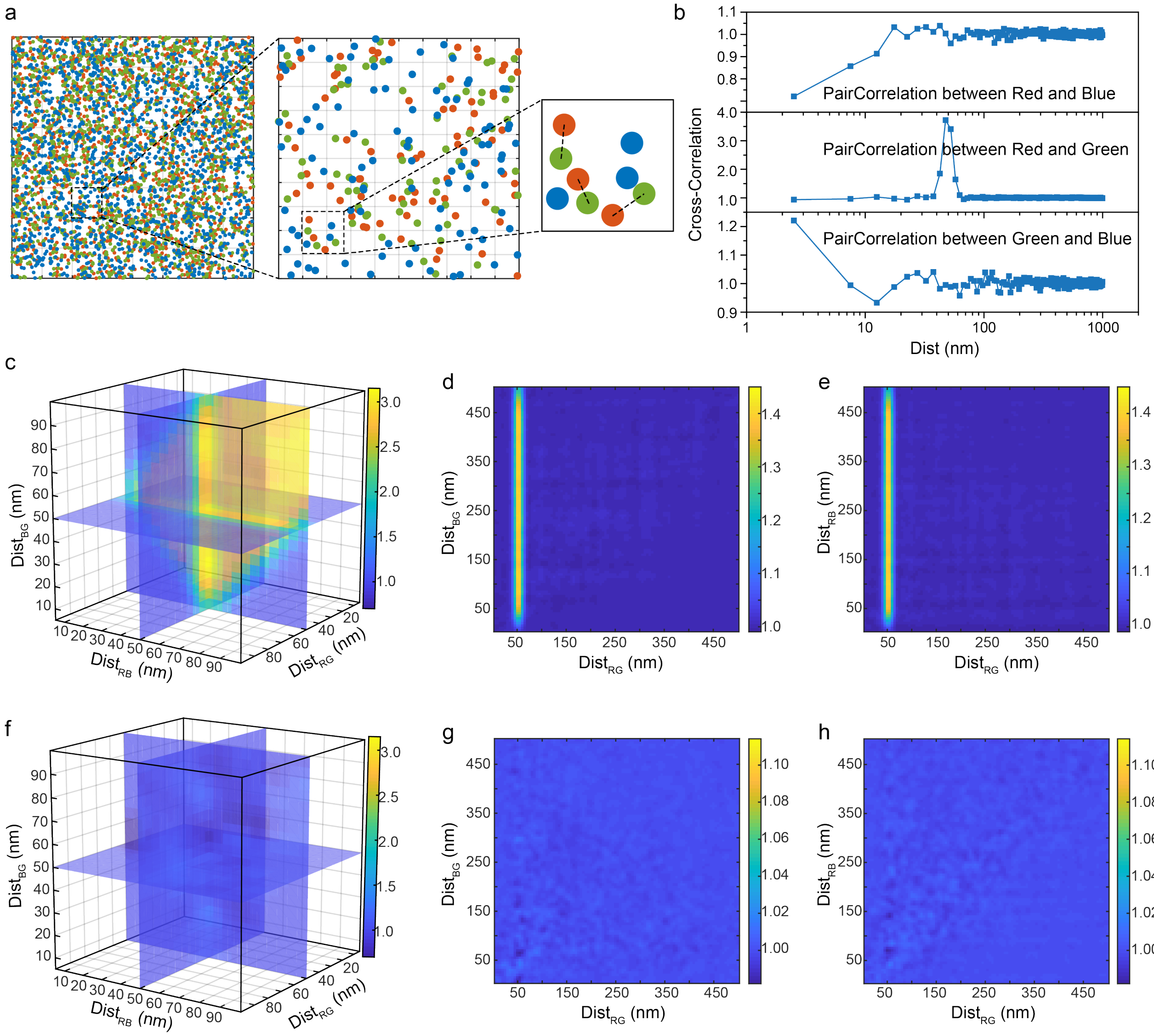

Supplementary Figure 2. | Difference between different forms of Triple-Correlation definitions $g(\mathbf{r}_{12}, \mathbf{r}_{13})$ and $g_\delta(\mathbf{r}_{12}, \mathbf{r}_{13})$. (**a**). simulated SMLM images where green and red are constantly correlated while blue is randomly distributed around. The colocalization amongst all three species occurs randomly and thus no significant $g_\delta(\mathbf{r}_{12}, \mathbf{r}_{13})$ is expected to be observed. (**b**). Pair-correlation between red and blue (top), red and green (middle), and green and blue (bottom), indicating the random distribution of the blue species. (**c**-**e**). Triple-correlation calculated defined as $g(\mathbf{r}_{12}, \mathbf{r}_{13})$ (c), and averaged (c) through different variables (d and e), displaying a strong pair-correlation signal. (**f**-**h**) Triple-correlation defined as $g_\delta(\mathbf{r}_{12}, \mathbf{r}_{13})$ (f), and averaged (f) through different variables (g and h), displaying no significant correlation among all three species.

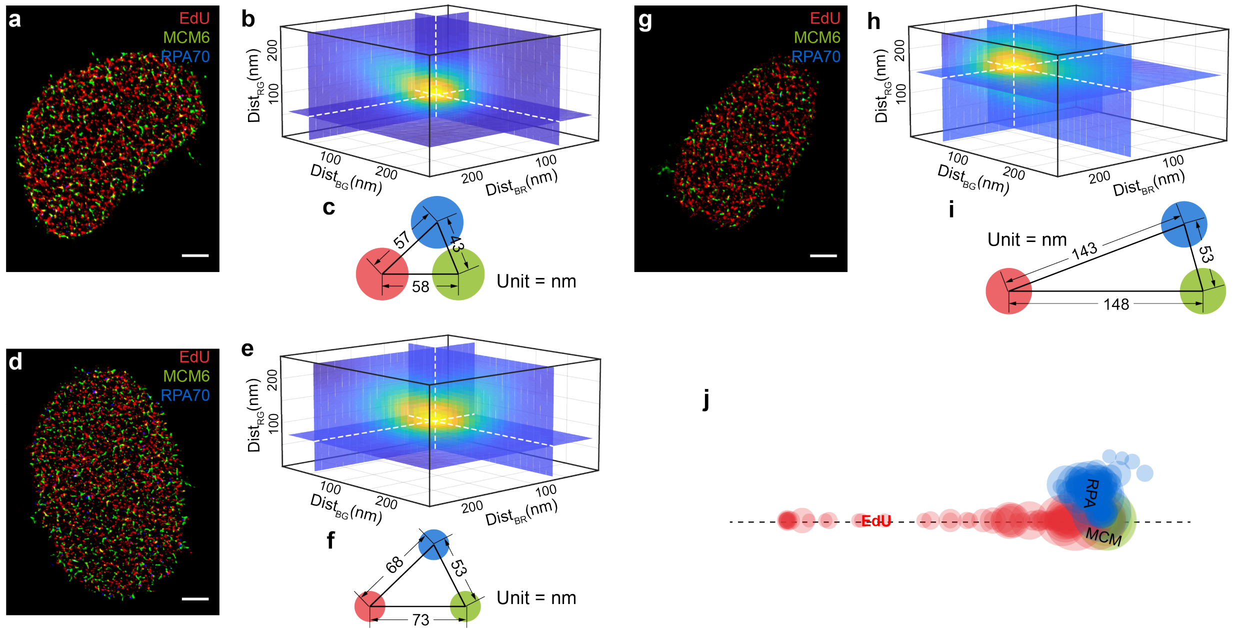

Supplementary Figure 3. | (**a**). A representative U2OS nucleus labeled with EdU (R), RPA (B), and MCM (G). The intrinsic configuration amongst RGB is barely seen by eye due to the highly dense complexity. (**b** and **c**). Triple-correlation (b) and the its reconstructed EdU-RPA-MCM configuration (c) of the nucleus in (a). (**d-i**). Two representative U2OS nuclei (d and g) and their triple-correlation profile. Scale bar = 2500 nm in (a), (d), and (g). (**j**). Configurations resolved from $N = 86$ nucleus are filtered by the criteria Dist_BG (distance between RPA and MCM) ≤ 100 nm and overlaid by aligning the RG edge onto the same horizontal, so that the relative distribution of RPA (B) to EdU (R) and MCM (G) is clearly seen to form an EdU-RPA-MCM sequential pattern. The circle size of at each vertex displays the triple-correlation amplitude. Source data for Supplementary Fig. 3j are provided as a Source Data file.

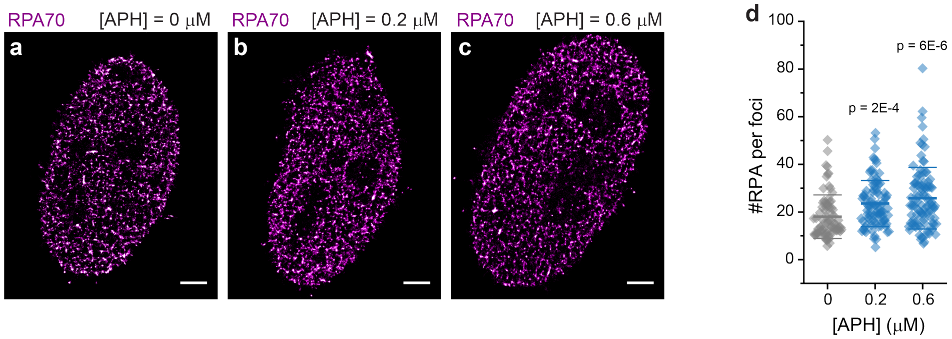

Supplementary Figure 4. | RPA accumulation at each replication foci upon Aphidicolin treatment. (**a**-**c**). Representative U2OS nuclei with RPA70 Immunol stained and treated by Aphidicolin of concentrations as indicated. Scale bar = 2500 nm. (**d**). Pair-Correlation analyses of the relative average amount of RPA within each replication focus. N = 79, 94, and 99 nuclei from [APH] = 0, 0.2μM, and 0.6 μM treatments, respectively, were submitted for Pair-Correlation analyses. The shown p-values are unpaired t-test of [APH] = 0 vs 0.2 μM, and [APH] = 0 vs 0.6 μM treatments. The Pair-Correlation analyses were performed following ref 12 in the main text. Source data for Supplementary Fig. 4d are provided as a Source Data file.

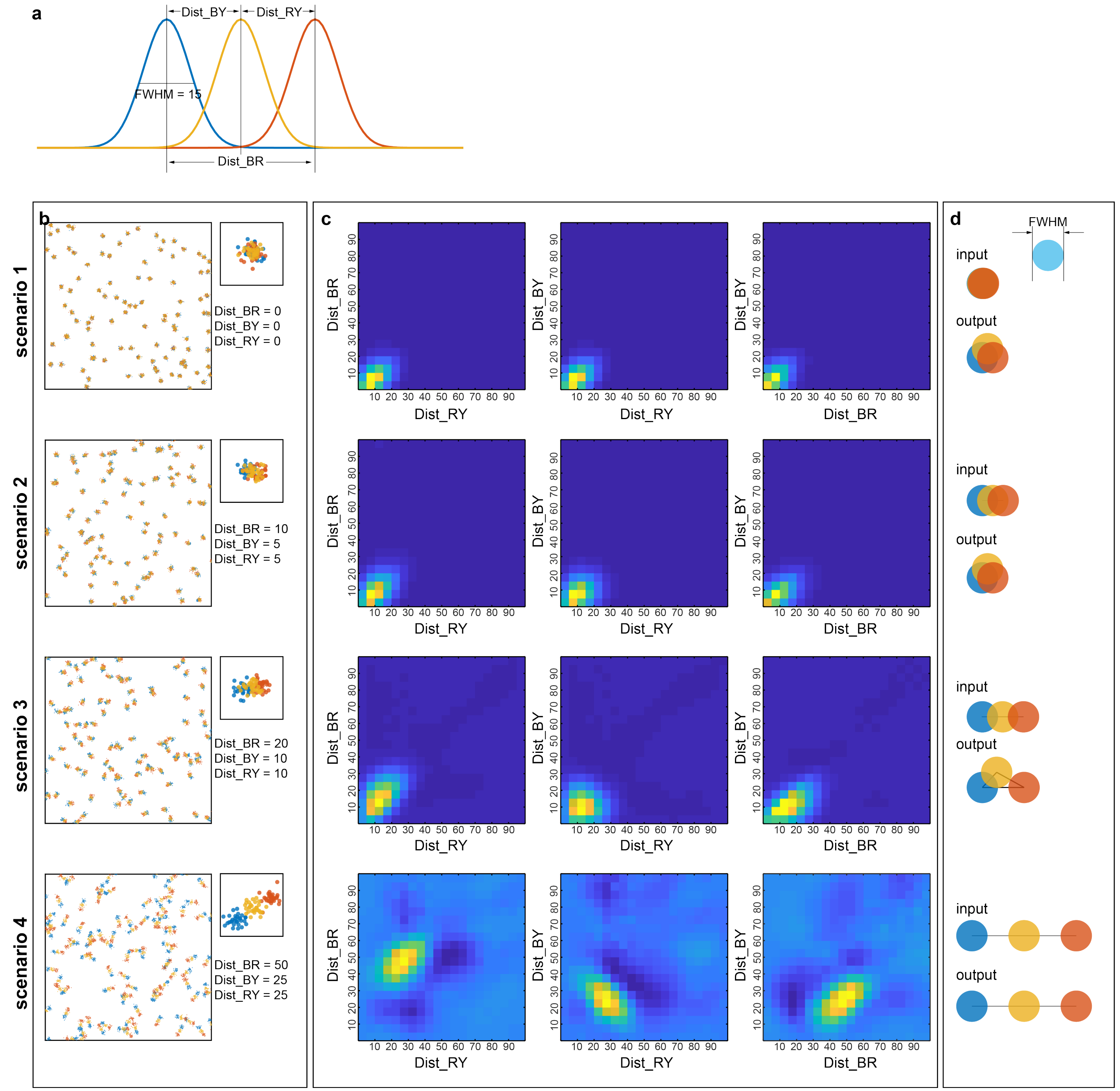

Supplementary Figure 5. | Evaluation of the Triple-Correlation function in resolving trimer configuration with a spacing comparable to SMLM localization precision. (**a**). Schematic illustration of the simulations. The blue, orange, and red "molecules" were linearly aligned with their pairwise center-to-center distance denoted as Dist_BY, Dist_BR, and Dist_RY for distances between Blue and Yellow, Blue and Red, and Red and Yellow, respectively. Around each "molecule", the localization coordinates were sampled from a 2D Gaussian distribution with its Full Width at Half Maxima (FWHM) = 15 AU, so that the experimental localization accuracy can be reflected in the simulated data (see Supplementary Methods). (**b**). Simulated three-color images with 4 different scenarios. The designed spacing of the trimers (Dist_BY, Dist_BR, and Dist_RY) were noted aside each simulated scene. (**c**). Triple-correlation profiles resulted from the different scenarios in (b). To better display the

Triple-Correlation results, the Triple-Correlation profile was decomposed into three 2D heatmaps in each row by averaging through each of the three variables to better display. (**d**). Schematic illustration of the input configurations as well as the Triple-Correlation resolved output configurations. As shown, the trimer configurations were not well resolved when the localization uncertainty is larger than its input spacing (scenario 1 and 2), but can be approximated when the spacing and the localization precision was comparable (scenario 3). The configurations were well resolved when the spacing is larger than the localization uncertainty (scenario 4). We note that besides the localization uncertainty in SMLM data, other experimental variable (e.g. insufficient labeling specificity, varying clustering of one or more of the examined three species, and etc.) can introduce additional complexity in Triple-Correlation based analyses, as well as other image analyses. As such the resolved triangular configurations serves as a range of relative mutual positioning or triple-wise colocalization of examined three species rather than as absolute measurements of spacing of the examined trimers.

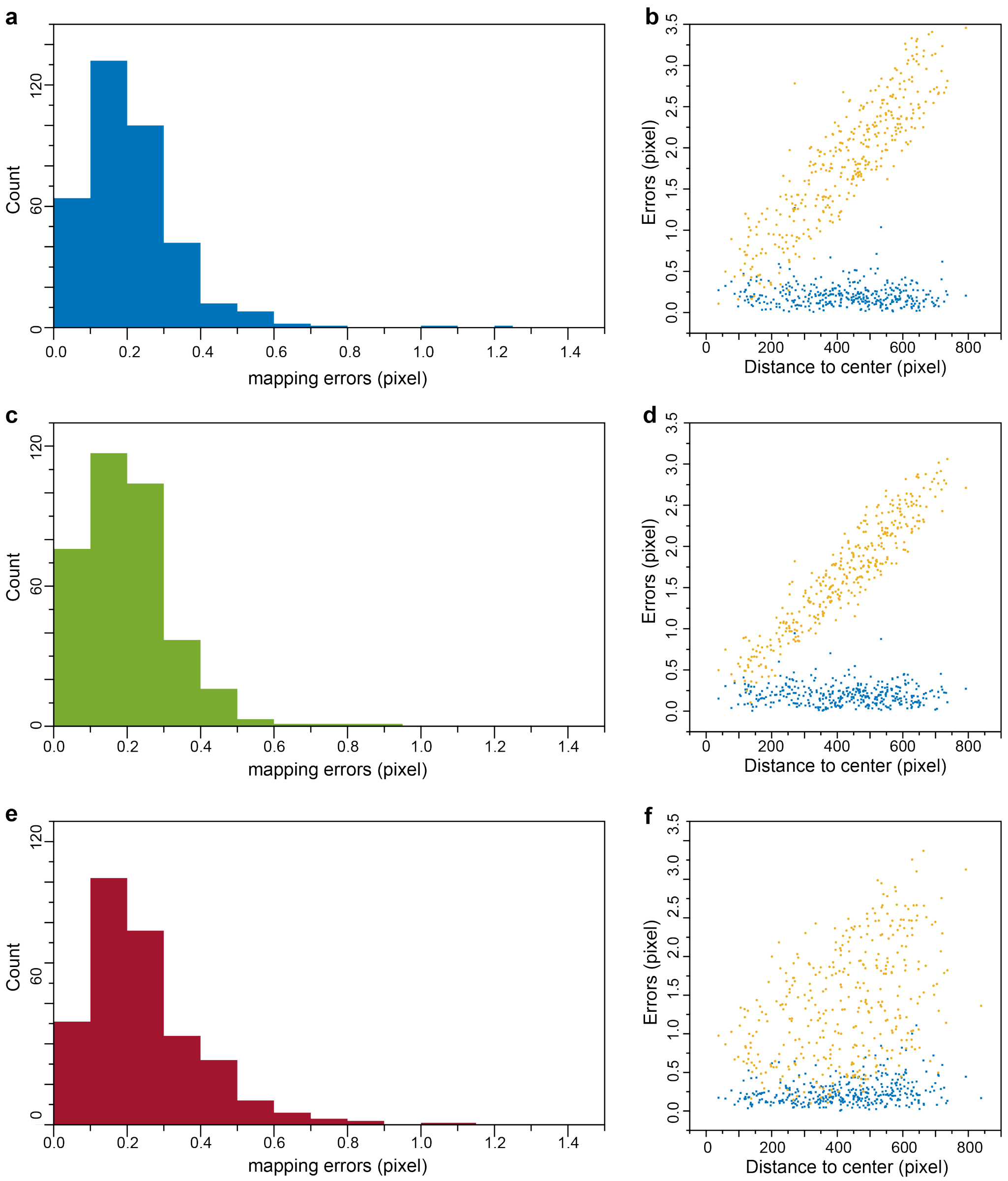

Supplementary Figure 6. | Evaluation of the Channel Alignments. (**a**, **c**, and **e**). Frequency counts of the alignment errors (1 pixel = 73.3 nm, Supplementary Methods) after polynomial mapping the Blue, Green, and Dark Red channels, respectively, on to the Red Channel. (**b**, **d**, and **f**) Plots of spatial errors between the Blue (b) / Green (d) / Dark Red (f) channel and the Red channel, as a function of the distance to the center of the imaging area. Errors before (yellow dots) and after (blue dots) the polynomial mapping indicates that the alignment strategy sufficiently reduced the chromatic aberrations. Source data are provided as a Source Data file.

Supplementary References

1 Ridgeway, W. K., Millar, D. P. & Williamson, J. R. The spectroscopic basis of fluorescence triple correlation spectroscopy. *J Phys Chem B* **116**, 1908-1919, (2012).

2 Nieuwenhuizen, R. P. *et al.* Measuring image resolution in optical nanoscopy. *Nat Methods* **10**, 557-562, (2013).

3 Veatch, S. L. *et al.* Correlation functions quantify super-resolution images and estimate apparent clustering due to over-counting. *PLoS One* **7**, e31457, (2012).
