# Supplementary material for: Ultrafast data mining of molecular assemblies in multiplexed high-density super-resolution images
Source: Nat Commun. 2019 Jan 10;10:119. doi: 10.1038/s41467-018-08048-2 (PMC6328550; doi:10.1038/s41467-018-08048-2)
Supplement: Supplementary file 4 — Reporting Summary [file 41467_2018_8048_MOESM4_ESM.pdf]

nature research

Corresponding author(s): Eli Rothenberg; Yandong Yin

Reporting Summary

Nature Research wishes to improve the reproducibility of the work that we publish. This form provides structure for consistency and transparency in reporting. For further information on Nature Research policies, see Authors & Referees and the Editorial Policy Checklist.

Statistical parameters

When statistical analyses are reported, confirm that the following items are present in the relevant location (e.g. figure legend, table legend, main text, or Methods section).

n/a	Confirmed	
☐	☒	The exact sample size (*n*) for each experimental group/condition, given as a discrete number and unit of measurement
☐	☒	An indication of whether measurements were taken from distinct samples or whether the same sample was measured repeatedly
☐	☒	The statistical test(s) used AND whether they are one- or two-sided<br>*Only common tests should be described solely by name; describe more complex techniques in the Methods section.*
☒	☐	A description of all covariates tested
☒	☐	A description of any assumptions or corrections, such as tests of normality and adjustment for multiple comparisons
☐	☒	A full description of the statistics including central tendency (e.g. means) or other basic estimates (e.g. regression coefficient) AND variation (e.g. standard deviation) or associated estimates of uncertainty (e.g. confidence intervals)
☐	☒	For null hypothesis testing, the test statistic (e.g. *F*, *t*, *r*) with confidence intervals, effect sizes, degrees of freedom and *P* value noted<br>*Give P values as exact values whenever suitable.*
☒	☐	For Bayesian analysis, information on the choice of priors and Markov chain Monte Carlo settings
☒	☐	For hierarchical and complex designs, identification of the appropriate level for tests and full reporting of outcomes
☒	☐	Estimates of effect sizes (e.g. Cohen's *d*, Pearson's *r*), indicating how they were calculated
☐	☒	Clearly defined error bars<br>*State explicitly what error bars represent (e.g. SD, SE, CI)*

Our web collection on statistics for biologists may be useful.

Software and code

Policy information about availability of computer code

Data collection	Experimental data were collected using Micro-Manager (v 1.4); Simulation data were generated via Matlab (v2017a)
Data analysis	Data analysis were performed mainly through C++ (via Intel Core i7 7800X) and CUDA8.0 (via NVIDIA GTX 1060); Data graphing were performed mainly through OriginLab(2016) and Matlab (v2017b).

For manuscripts utilizing custom algorithms or software that are central to the research but not yet described in published literature, software must be made available to editors/reviewers upon request. We strongly encourage code deposition in a community repository (e.g. GitHub). See the Nature Research guidelines for submitting code & software for further information.

Data

Policy information about availability of data

All manuscripts must include a data availability statement. This statement should provide the following information, where applicable:
- Accession codes, unique identifiers, or web links for publicly available datasets
- A list of figures that have associated raw data
- A description of any restrictions on data availability

A reporting summary for this Article is available as a Supplementary Information file. The major source data underlying Figs 2c, 3j, and 4d-g and Supplementary Figs 3j, 4d, and 6 are provided as a Source Data file. Other simulated and experimental data is available from the authors upon requests.

Field-specific reporting

Please select the best fit for your research. If you are not sure, read the appropriate sections before making your selection.

☒ Life sciences ☐ Behavioural & social sciences ☐ Ecological, evolutionary & environmental sciences

For a reference copy of the document with all sections, see nature.com/authors/policies/ReportingSummary-flat.pdf

Life sciences study design

All studies must disclose on these points even when the disclosure is negative.

Sample size	The sample size were not considered here. The presented algorithms provided a new method for triple-correlation computation, and in a sense, the sample size could be considered to be the number of ROIs that submitted to such analyses. However, these are governed by the studies in which the algorithms are applied other than the algorithms themselves for statistical consideration. The sample sizes given in this manuscripts were sufficient as considerations of experimental validation.
Data exclusions	Geometric configuration resolved by Triple-Correlation in Figure 3 and Figure 4 were filtered by the resolved distance between PCNA and MCM (< 100 nm), which could be used as criteria for single-fork identification. Note that the resolved distance between PCNA and MCM is a averaged distance that convolves the actual averaged distance, apparent size of PCNA and MCM that involves their localization accuracy, and multi-color mapping errors.
Replication	The statistics given in the main text were analyzed through 3 experimental replicates for both pair- and triple-correlation analyses.
Randomization	Randomizations were not relevant to the presented algorithms.
Blinding	In order to characterize DNA replication, only PCNA-positive (S-phase indicator) U2OS cells were collected and analyzed.

Reporting for specific materials, systems and methods

Materials & experimental systems

n/a	Involved in the study
☒	☐ Unique biological materials
☐	☒ Antibodies
☐	☒ Eukaryotic cell lines
☒	☐ Palaeontology
☒	☐ Animals and other organisms
☒	☐ Human research participants

Methods

n/a	Involved in the study
☒	☐ ChIP-seq
☒	☐ Flow cytometry
☒	☐ MRI-based neuroimaging

Antibodies

Antibodies used	anti-PCNA antibody (Abcam, ab201672); anti-MCM antibody (Abcam, ab211916); anti-RPA antibody (Abcam, ab79398 and ab199240) ; goat anti-Rabbit secondary antibody (ThermoFisher, A-21039)
Validation	The anti-PCNA antibody (PC10) was validated by Hampp S et al. DNA damage tolerance pathway involving DNA polymerase ? and the tumor suppressor p53 regulates DNA replication fork progression. Proc Natl Acad Sci U S A 113:E4311-9 (2016). and at least other 212 publications in various application including IF.<br>The anti-MCM antibody (EPR17686) was validated by Wu R et al. H3K9me3 demethylase Kdm4d facilitates the formation of pre-initiative complex and regulates DNA replication. Nucleic Acids Res 45:169-180 (2017). In applications of ChIP;<br>The anti-RPA antibodiy (EPR3472, ab79398) was validated by Toledo LI et al. ATR prohibits replication catastrophe by preventing global exhaustion of RPA. Cell 155:1088-103 (2013). In applications of WB and IF.<br>The anti-RPA antibody(EPR3472, ab199240) is the same antibody as ab79398 but with Alexa Fluor 647 directly conjugated.

Eukaryotic cell lines

Policy information about cell lines

Cell line source(s)	U2OS cell lines were obtained from ATCC

Authentication	No cell line authentication was performed
Mycoplasma contamination	No Mycoplasma contamination test was performed
Commonly misidentified lines (See ICLAC register)	No misidentified lines were used
